# Optimization of Xylanase Production from *Penicillium* sp.WX-Z1 by a Two-Step Statistical Strategy: Plackett-Burman and Box-Behnken Experimental Design

**DOI:** 10.3390/ijms130810630

**Published:** 2012-08-23

**Authors:** Fengjie Cui, Liming Zhao

**Affiliations:** 1 School of Food and Biological Engineering, Jiangsu University, Zhenjiang 212013, China; E-Mail: fengjiecui@163.com; 2 Department of Respiratory Disease, Changzheng Hospital, Second Military Medical University, 415 Fengyang Road, Shanghai 200003, China

**Keywords:** *Penicillium* sp.WX-Z1, xylanase, nutrient sources, Plackett-Burman design, Box-Behnken design

## Abstract

The objective of the study was to optimize the nutrition sources in a culture medium for the production of xylanase from *Penicillium* sp.WX-Z1 using Plackett-Burman design and Box-Behnken design. The Plackett-Burman multifactorial design was first employed to screen the important nutrient sources in the medium for xylanase production by *Penicillium* sp.WX-Z1 and subsequent use of the response surface methodology (RSM) was further optimized for xylanase production by Box-Behnken design. The important nutrient sources in the culture medium, identified by the initial screening method of Placket-Burman, were wheat bran, yeast extract, NaNO_3_, MgSO_4_, and CaCl_2_. The optimal amounts (in g/L) for maximum production of xylanase were: wheat bran, 32.8; yeast extract, 1.02; NaNO_3_, 12.71; MgSO_4_, 0.96; and CaCl_2_, 1.04. Using this statistical experimental design, the xylanase production under optimal condition reached 46.50 U/mL and an increase in xylanase activity of 1.34-fold was obtained compared with the original medium for fermentation carried out in a 30-L bioreactor.

## 1. Introduction

Xylanases have been widely used for clarifying fruit juices and wine [1–3], in food processing in combination with cellulases [4,5], bleaching in the paper and pulp industry [6–8], and hydrolyzing agricultural waste to produce renewable energy products in the biofuel industry [9,10]. In general, the production of xylanase based on microbial fermentation and biosynthesis renders its industrial application more feasible and economical. Not surprisingly, much research has been carried out to make the production of enzymes more cost effective. Filamentous fungi have been widely used to produce hydrolytic enzymes for industrial application, including xylanases, whose levels in fungi are generally much higher than in yeast and bacteria. The most common industrial xylanase producing strains are the species *Aspergillus* sp. and *Trichoderma* sp., as well as bacterial strains of the species *Bacillus* sp. [11].

Two possible cultivation methods for microbial xylanase production are solid-state and submerged cultivation. Nowadays, the submerged cultivation is used more widely, allowing a higher degree of processes intensification and a better level of automation. Xylanases from filamentous fungi are extra-cellular, inducible enzymes. The nutrient medium compositions and culture factors strongly influence the xylanase production. For example, sodium nitrate and yeast extract resulted in higher xylanase production by *Penicillium oxalicum* in comparison to other nitrogen sources [12]. However, some other important nitrogen sources such as trypton and casein were not investigated in their research. Amino acids and their analogues are also known to simulate the production of enzymes, such as α-amylase, β-galactosidase and xylanase. dl-2-amino-*n*-butyric acid could enhance total xylanase production from *Staphylococcus* sp. SG-13 up to 5.5-fold [13], and the combination of dl-norleucine, l-leucine and dl-isoleucine also could synergistically simulate xylanase production [14]. Therefore, optimization of fermentation conditions and screening of important nutritional factors are of essential importance to determine the optimal parameters for efficient production. As a result, the production cost for xylanase should be significantly reduced.

Conventional single dimensional search involves changing one independent variable at a time while fixing the others at a constant level, which gives unreliable results, inaccurate conclusion, and even frequent interactions of two or more factors. Statistical experimental designs including Plackett-Burman and response surface methodologies (RSM) can collectively eliminate these limitations of a single factor optimization process. Plackett-Burman design [15] is a powerful statistical technique for screening medium components in a shake flask and has been widely used in fermentation optimization [16–19]. This technique cannot determine the exact quantity but can provide indication and tendency regarding the necessity of each factor in relatively few experiments. The following response surface methodology (RSM) can provide mathematical models showing the dependence of the enzyme activity on independent variables (the concentration of the separate components of the nutrient medium or operating parameters), and even give predictive results of responses and the possible levels of related independent variables.

The genus *Penicillium* is a promising producer of xylanolytic enzymes suitable for applications in various industries. *Penicillium* sp.WX-Z1, an acidic xylanase producer, was initially isolated from decaying agricultural waste and stored in our laboratory. Primary studies in terms of characterization of the enzyme suggested that high xylanase activity was favored with low pH conditions, indicating its potential industrial applications. However, the low production yield limits the potential application for various industrial purposes. Therefore, the objectives of this paper were to select the important nutrient sources by Plackett-Burman design and determine their optimal concentrations in the culture medium using a factorial Box-Behnken design methodology that can substantially increase the xylanase production, and could be verified in a 30-L bioreactor fermentation.

## 2. Results

### 2.1. Screening of Important Nitrogen Sources for Xylanase Production by *Penicillium* sp.WX-Z1

As shown in Table 1, the maximum and minimum levels of variables chosen for trials in Plackett-Burman experimental design represent a big variation in nutrient sources. The design for 40 trials with two levels of concentrations for each variable with the resultant enzyme activities are shown in Table 2. The variables (*X*_1_–*X*_19_) represent 19 different substrates, organic and inorganic nitrogen sources, trace elements, and other affecting factors. The results of statistical analysis shown in Table 3 suggested that the confidence levels of *X*_1_ (Wheat bran), *X*_4_ (Yeast extract), *X*_8_ (NaNO_3_), *X*_13_ (MgSO_4_), and *X*_14_ (CaCl_2_) were 99.33%, 96.56%, 97.93%, 95.54% and 95.15%, respectively, which are considered to significantly influence xylanase production. Other independent variables with confidence levels below 95% were generally considered insignificant. After the first optimization, the nutrient sources were reduced to five major variables by the Plackett-Burman experimental design, suggesting that Plackett-Burman design is a powerful tool for screening fermentation factors. The exact optimal values of the individual factors were still unknown but could be determined by the subsequent Box-Behnken design.

### 2.2. Optimization of Screened Nutrient Sources for Xylanase Production by *Penicillium* sp.WX-Z1 Using Response Surface Methodology

Five variables of *X*_1_ (Wheat bran), *X*_4_ (Yeast extract), *X*_8_ (NaNO_3_), *X*_13_ (MgSO_4_), and *X*_14_ (CaCl_2_) were chosen for further study based on Box-Behnken design. Table 4 shows the 46 experimental runs with different combinations of the five variables along with experimental responses. As shown in Table 4, a considerable variation in the xylanase production was found, depending on the levels of the five variables in the medium. The maximum xylanase production was found to be 44.32 U/mL in run number 23 and the minimum 8.52 U/mL in run number 1. For estimation of the RSM model errors, the centre point in the design was repeated six times.

By applying multiple regression analysis on the experimental data, the following second order polynomial equation was found to explain the xylanase production by only considering the significant terms and is shown below:

(1) $$\begin{array}{c} Y=38.80+12.59X_{1}+2.76X_{2}+4.78X_{3}+1.26X_{4}+\\ 1.45X_{5}-7.55{X_{1}}^{2}-5.29{X_{2}}^{2}-4.06{X_{3}}^{2}-4.96{X_{4}}^{2}-\\ 4.73{X_{5}}^{2}-2.69X_{1}X_{2}-3.25X_{1}X_{4}-2.50X_{1}X_{5} \end{array}$$

where *Y* is the predicted response; *X*_1_, *X*_2_, *X*_3_, *X*_4_, *X*_5_ are coded values of wheat bran, yeast extract, NaNO_3_, Mg and Ca, respectively.

Analysis of variance (*F*-test) showed that the second model is well adjusted to the experimental data. The coefficient of variation (CV) indicates the degree of precision with which the treatments are compared. The lower value of CV (7.74) indicates a greater reliability in the experiments performed. The determination coefficient (*R*^2^), which is commonly used to check the goodness of the model, implies that the sample variation of 96.7% for xylanase production is attributed to the independent variables in the current study. The value of *R* (0.9834) for Equation 1 being near to 1 indicates a close agreement between the experimental results and the theoretical values predicted by the model equation. Linear, cross product and quadratic terms were significant at the 1% level. Therefore, the quadratic model was selected in this optimization study. Table 5 shows the Student *t*-distribution, the corresponding *P*-value, and the parameter estimate.

Three-dimensional response plots and their corresponding contour plots were drawn on the basis of the model equation, to investigate the interaction among the variables and to determine the optimum concentration of each factor for maximum xylanase production by *Penicillium* sp.WX-Z1. The contour plots affirm that the objective function is unimodal in nature which show an optimum at the boundaries.

**Figure 1:**
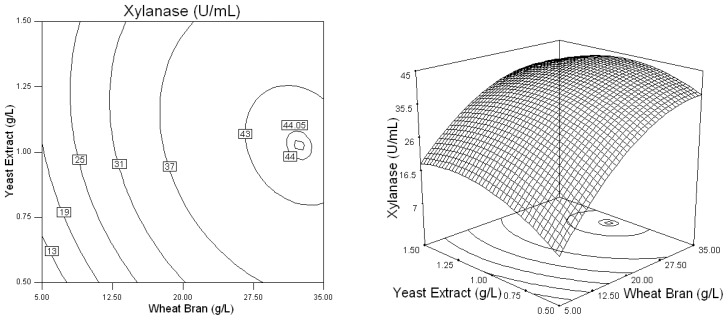
Response surface plot and contour plot of the combined effects of wheat bran and yeast extract on xylanase production by *Penicillium* sp.WX-Z1.

**Figure 2:**
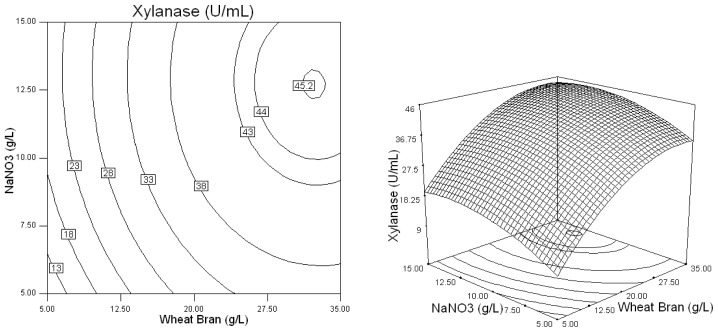
Response surface plot and contour plot of the combined effects of wheat bran and NaNO_3_on xylanase production by *Penicillium* sp.WX-Z1.

**Figure 3:**
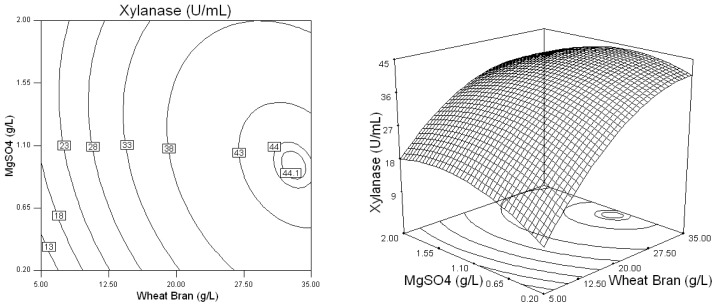
Response surface plot and contour plot of the combined effects of wheat bran and MgSO_4_ on xylanase production by *Penicillium* sp.WX-Z1.

The effect of varying the concentration of *X*_1_ (Wheat bran) and one of the other variables is shown in Figures 1–3. It can be seen from Figure 1 that, xylanase production tends to increase while gradually increasing the value of wheat bran concentration.

From Figure 2, which shows the effects of *X*_1_ (Wheat bran) and *X*_3_ (NaNO_3_) on xylanase production by *Penicillium* sp.WX-Z1, it was obvious that when wheat bran was at a low level, the effect of NaNO_3_ was negligible. When the wheat bran concentration was at a higher level, xylanase production steadily increased with increasing NaNO_3_ up to 0.3–0.9 (coded value), but decreased slowly beyond this range.

**Figure 4:**
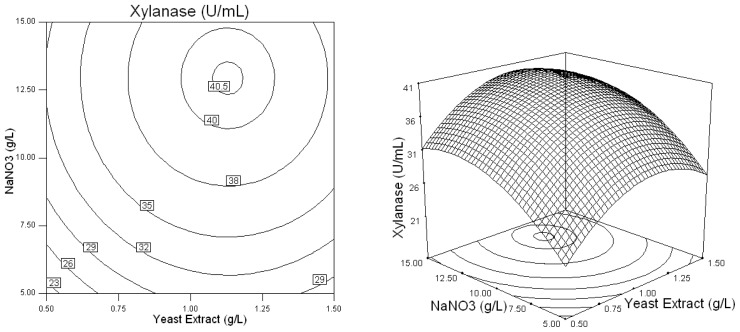
Response surface plot and contour plot of the combined effects of yeast extract and NaNO_3_ on xylanase production by *Penicillium* sp.WX-Z1.

**Figure 5:**
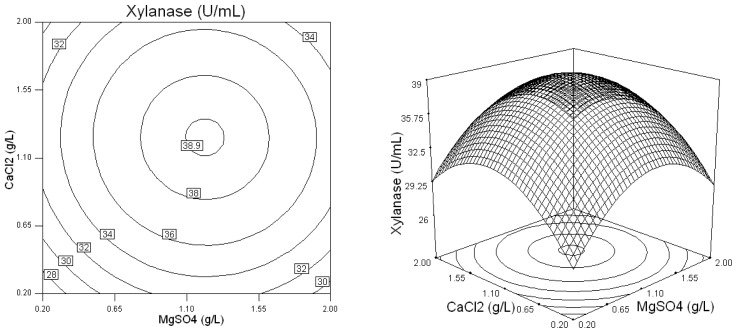
Response surface plot and contour plot of the combined effects of MgSO_4_ and CaCl_2_ the on xylanase production by *Penicillium* sp.WX-Z1.

Figure 4 describes the effects of *X*_3_ (NaNO_3_) and *X*_4_ (MgSO_4_) on xylanase production and indicates when *X*_3_ (NaNO_3_) and *X*_4_ (MgSO_4_) were in the ranges of 0.3 to 0.8 (coded value), and −0.1 to 0.3 (coded value), the xylanase activity was higher than 40 U/mL. Figure 5 shows the effects of *X*_4_ (MgSO_4_) and *X*_5_ (CaCl_2_) on xylanase production. Evidently, the higher level of the two variables in the medium ensured more xylanase production than 39 U/mL. Thus a certain quantity of trace elements, of which MgSO_4_ and CaCl_2_ were identified to be significant for *Penicillium* sp.WX-Z1, must be added in order to obtain the higher xylanase production.

**Figure 6:**
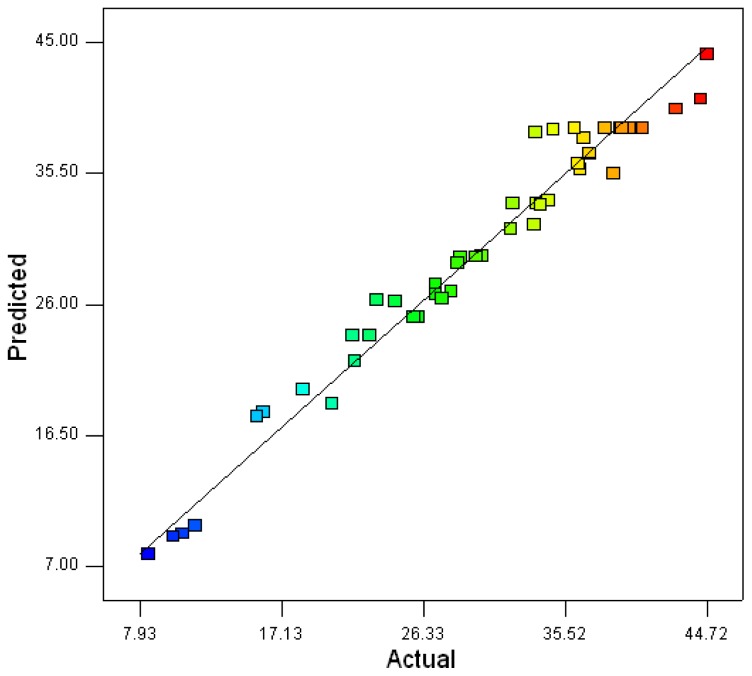
Observed xylanase activity *versus* the predicted xylanase activity under the optimum fermentation conditions.

The statistical optimal values of variables are obtained when moving along the major and minor axis of the contour and the response at the centre point yields the maximum xylanase production. These observations were also verified from canonical analysis of the response surface. Canonical analysis revealed a minimum region for the model. The stationary point presenting maximum xylanase had the following critical values (g/L): wheat bran, 32.8; yeast extract, 1.02; NaNO_3_, 12.71; MgSO_4_, 0.96; CaCl_2_, 1.04. The predicted xylanase activity for these conditions was 45.38 U/mL. The observed xylanase activity *versus* the predicted xylanase activity under optimum fermentation conditions is shown in Figure 6. A repeat fermentation of xylanase by *Penicillium* sp.WX-Z1 under optimal condition was carried out to verify the optimization. The maximum xylanase level obtained was 46.50 U/mL, which was close to the predicted value.

### 2.3. 30-L Bioreactor Fermentation Results

**Figure 7:**
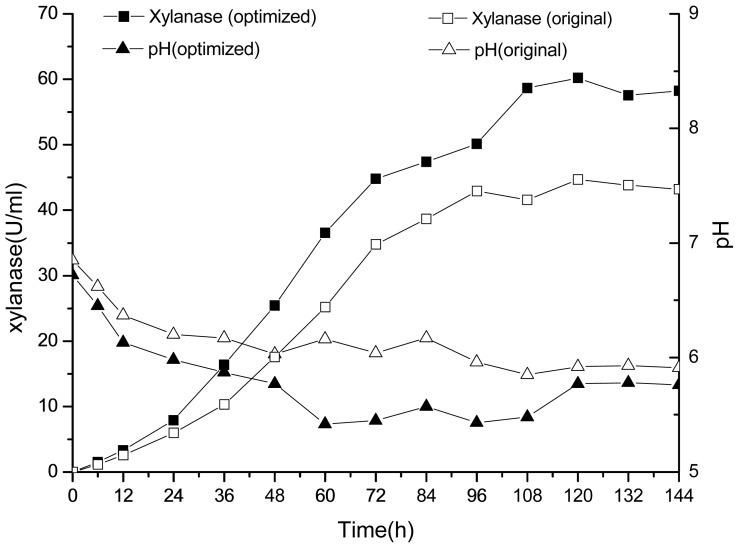
Time courses of xylanase production (**box**) and pH (**triangle**) during 30-L bioreactor fermentation by *Penicillium* sp.WX-Z1 under optimized (**filled**) and original (**open**) conditions.

Figure 7 shows the comparison of xylanase production from optimized and original media compositions by *Penicillium* sp.WX-Z1 in a 30-L bioreactor. The time courses of xylanase production and pH value changes under optimized and original conditions were similar, and xylanase increased slowly during the first 36-h fermentation, and then entered the log phase after 36 h. Xylanase reaches a peak value of 60.23 U/mL after 120-h fermentation with *Penicillium* sp.WX-Z1 under the optimized conditions, while a maximum level of 44.7 U/mL was observed under the original conditions. Xylanase activity began to fluctuate after 120 h. Compared with xylanase fermentation under the original conditions, an increase in xylanase activity of 1.34-fold was obtained by optimizing the media compositions.

## 3. Discussion

Xylanolytic enzymes from microorganism have attracted a great deal of attention in the last decade particularly due to their biotechnological potential in various industrial processes. Although previous researches regarding acidic xylanase purification and characterization have been reported, little information on the optimization of its production using *Penicillium* species is available. In this paper, a two-step statistical strategy combining Plackett-Burman with Box-Behnken Experimental Design was applied to find the key parameters and their optimized levels for xylanase production from *Penicillium* sp.WX-Z1.

*Penicillium* sp.WX-Z1 achieved the maximum xylanase production of 29.72 U/mL when the variables were at a maximum level in Plackett-Burman design which was higher than our previous isolated strain *Penicillium* with a verified optimal xylanase activity of 14.50 U/mL (Table 2) [20]. Hence, *Penicillium* sp.WX-Z1 is a competitive producer of acidic xylanase even compared to other species. For example, *Bacillus circulans* had the maximum xylanase production of 19.1 U/mL after optimization with a 3^3^ factorial response surface design [21].

Based on the results of Plackett-Burman design, five variables of Wheat bran, Yeast extract, NaNO_3_, MgSO_4_, and CaCl_2_ were chosen for further optimization by using the Box-Behnken design with 46 experimental runs. From Tables 4 and 5, a second order polynomial equation was obtained to describe the xylanase production with a determination coefficient (*R*^2^) of 96.7% and linear, cross product and quadratic terms significant at the 1% level, indicating that all of the five independent variables had significant effects on xylanase production. The 3D response surface and the contour plots described by the regression model were drawn to illustrate the effect of these five independent variables, and the interactive effects of each independent variable on the response/variables (Figures 1–5). For example, Figure 1 shows that xylanase production tends to increase with gradually increasing value in the substrate wheat bran concentration. Simultaneously, it was highlighted that *Penicillium* sp.WX-Z1 could make better use of wheat bran to produce more xylanase, which agrees well with the report by Beg *et al.* [14] that wheat bran could effectively induce higher xylanase production by *Streptomyces* sp. QG-11-3. Li *et al.* [22] also reported the importance of the substrate concentration for xylanse production by *Aspergillus awamori*. These facts might be accounted for by the report that the enzymes involved in substrate degradation were generally inducible and were formed only when the corresponding substrate was present in the nutrient solution. Liu *et al.* [23] and other researchers [24] reported xylose could induce xylanase production by *Trichosporon cutaneum* SL409 and *Aspergillus nidulans*, respectively. Xylanase production by *Trichoderma reesei* was found to depend on the concentration of glucose in the media [25] but was restrained by *Aspergillus nidulans* [24] and by *Penicillium* sp.40 [26]. Figure 1 shows the shape of the contour of wheat bran against yeast extract, indicating that a high yeast extract level has a high contribution to the yield of xylanase. This is similar to the conclusion reported by Lemos *et al.* [12] that increasing yeast extract concentration in the medium was beneficial to xylanase production by *Aspergillus awamori* NRRL 3112 and that ions in the yeast extract may play an important role for the metabolic enzyme. It was also noted from Figure 1a that, when wheat bran was in the range of 0.7 to 0.95 (coded value) and yeast extract in the range of −0.1 to 0.25 (coded value), the xylanase production was higher than 44 U/mL. Figure 2 shows the effects of *X*_1_ (Wheat bran) and *X*_3_ (NaNO_3_) on xylanase production by *Penicillium* sp. WX-Z1 and obviously the effect of NaNO_3_ was negligible when the wheat bran level was low. Not surprisingly, as an inorganic nitrogen source, NaNO_3_ plays an important part in xylanase production with different microorganisms [27]. From Figure 3, the xylanase production could achieve a higher activity than 44 U/mL when the concentration of *X*_4_ (MgSO_4_) was at a higher level between −0.3 and 0.1 (coded value). It is possible that Mg^2+^ has a positive effect on the stabilization of the ribosome and cellular membranes and Ca^2+^ plays a protector role in the medium, which relatively enhances the activity of xylanase. Although ions Mg, Ca, Fe, Mn and Zn were chosen in the Plackett-Burman design, the result showed that only Ca^2+^ and Mg^2+^ had a significant impact on xylanase production. Some researchers have suggested the importance of Mg^2+^ as a trace element for xylanase production by *Aspergillus fischeri* Fxn 1 [28] and by *Streptomyces olivaceoviridis* E-86 [29], and other researchers found that Mg^2+^ combined with Fe^2+^ played an important part in xylanase production by *Streptomyces* sp. QG-11-3 [14], and that Zn^2+^, Mg^2+^ combining with Fe^2+^ could stimulate the yield of xylanase by *Streptomyces thermodiasticus* [30].

The canonical analysis revealed a maximum xylanase activity of 46.50 U/mL under the optimal conditions of wheat bran, 32.8 g/L; yeast extract, 1.02 g/L; NaNO_3_, 12.71 g/L; MgSO_4_, 0.96 g/L and CaCl_2_, 1.04 g/L. Validation experiments were also carried out to verify the availability and the accuracy of the models, and the results showed that the predicted values agreed well with the experimental values. The batch fermentation results in a 30-L fermenter also showed that optimized culture medium could improve the production of xylanase from 60.23 U/mL from *Penicillium* sp.WX-Z1 in a large-scale fermentation process. It suggests that the production of acidic xylanase by wild-strain alkaliphilic *Penicillium* sp.WX-Z1 may be interesting for industrial applications.

## 4. Experimental Section

### 4.1. Microorganism

The *Penicillium* sp.WX-Z1 strain was isolated from decaying agricultural waste and stored at 4 °C on potato dextrose agar (PDA) in our laboratory. Spore suspensions were made from six-day-old cultures that had been grown on PDA slopes at 30 °C. Sterile distilled water was aseptically added to each slope and a suspension of the spores made by lightly brushing the mycelium with a sterile wire loop. The suspension, where necessary, was diluted with sterile distilled water to give a final spore count of 1 × 10^7^ spores/mL.

### 4.2. Medium and Cultivation

The medium used for xylanase production was composed of (g/L) [20]: wheat bran 50; glucose 10; KH_2_PO_4_ 1; MgSO_4_·7H_2_O 1; CaCl_2_·2H_2_O 0.3; at pH 5.0. Nitrogen sources were added according to the statistical experimental design. The microorganism was cultured in 75 mL of medium in 250 mL Erlenmeyer flasks and incubated at 30 °C on a rotary shaker (150 rpm). After 96 h fermentation, the mycelium was separated from the enzyme-containing broth by centrifugation at 10,000× *g* for 15 min to obtain the crude enzyme preparation.

### 4.3. Optimization Procedure

The optimization of the nitrogen source in the medium constituents for xylanase production by *Penicillium* sp.WX-Z1 was carried out in two stages.

#### 4.3.1. Plackett-Burman Design for Identification of Important Nutrients

Plackett-Burman design based on the first order model:

(2) $$Y=\beta_{0}+\sum \beta_{i}x_{i}$$

This was used to screen the important variables that influence xylanase production. The total number of trials to be carried out according to Plackett-Burman is *k* + 1 where *k* is number of variables (medium components). Each variable is represented at two levels, high and low which are denoted by (+1) and (−1), respectively. The coded level of each variable is shown in Table 1 and the experimental design with 40 trials for screening 19 variables is shown in Table 2. All experiments were performed in duplicate and the mean values are given. The variables, whose confidence levels are greater than 95% in Table 3, were considered to significantly influence xylanase production.

The standard error (S. E.) of the concentration effect was the square root of the variance of an effect and the significant level (*p*-value) of each concentration effect was determined using Student’s *t*-test:

(3) $$t_{xi}=\frac{E(X_{i})}{\text{S}.\text{E}.}$$

where *E*(*X_i_*) is the effect of variable *X_i_*.

#### 4.3.2. Box-Behnken Design

Response surface methodology (RSM) was used to optimize the screened components for enhancing xylanase production. A Box-Behnken factorial design [31] with five factors and three levels, including six replicates at the centre point, was used for fitting a second order response surface. Table 4 gives the factors and their values, and the experimental design, respectively. This methodology allows the modeling of a second order equation that describes the process. Xylanase production was analyzed by multiple regression through the least squares method to fit the following equation:

(4) $$Y=\beta_{0}+\sum \beta_{i}x_{i}+\sum \beta_{ij}x_{i}x_{j}+\sum \beta_{ii}x_{i}^{2}$$

where *Y* is the measured response variable; *β*_0_, *β_i_*, *β_ij_*, *β_ii_* are constant and regression coefficients of the model, and *x_i_*, *x_j_* represent the independent variables (enzymatic hydrolysis parameters) in coded values.

Data from the Box-Behnken factorial design for the optimization of xylanas production was subjected to a second-order multiple regression analysis using the least squares regression methodology to obtain the parameter estimators of the mathematical model. The regression analysis and analysis of variance (ANOVA) were carried out using the RSREG procedure of the SAS statistical package (version 8.1, SAS Institute, Cary, NC, USA, 2000) to fit second order polynomial equations for all response variables. Canonical analysis, which is used to predict the shape of the curve generated by the model, was carried out as well. Response surface was made by the fitted quadratic polynomial equation obtained from RSREG analysis, holding independent variables with two parameters at a constant value and changing the other two variables.

### 4.4. Bioreactor Cultivation Conditions

Bioreactor experiments were carried out in a 30-L bioreactor (18 L working volume) equipped with instruments and controllers for agitation, temperature and pH. Fermentor contents were stirred at 90–120 rpm to ensure homogeneous mixing and the air-flow rate was controlled at 0.5 vvm, temperature was maintained at 30 °C. The fermentation process was maintained for 144 h. Samples were collected at various intervals (6 h) from the bioreactor for analysis.

### 4.5. Analytical Method

The xylanase activity was determined according to our previous method by measuring the release of reducing sugars from oat spelt xylan (1%, *w*/*v*) using the dinitrosalicylic acid method [32]. Reaction mixture containing 2 mL of a solution of 1% oat spelt xylan in citrate buffer 50 mM, pH 5.0 plus 1 mL of the diluted crude enzyme, was incubated for 30 min at 50 °C. One unite of xylanase was defined as the amount of enzyme required to released 1 μmol of xylose from xylan in 1 min under the assay condition.

## 5. Conclusions

Statistical optimization of the nutrient sources in a medium using Plackett-Burman and Box-Behnken design appears to be a valuable tool for the production of xylanase by *Penicillium* sp.WX-Z1. Plackett-Burman design made it possible to consider a large number of variables and avoid the loss of information, which might be essential in the optimization of the fermentation process. Wheat bran, yeast extract, NaNO_3_, MgSO_4_, and CaCl_2_ were chosen as the nutrient sources for xylanase production by *Penicillium* sp.WX-Z1 in the Plackett-Burman design. Their concentrations in the medium were further optimized by Box-Behnken design. The predicted and verifiable xylanase activities under optimal conditions were 45.38 U/mL and 46.50 U/mL, respectively. An increase in xylanase activity of 1.34-fold was also obtained by adjusting the medium compositions for fermentation carried out in a 30-L bioreactor.

## Figures and Tables

**Table 1:** Variables and their levels employed in Plackett-Burman design for screening of culture conditions affecting xylanase production by *Penicillium* sp.WX-Z1.

Variable code	Variable	Value	

		−1	+1
*X*_1_	Wheat bran (g/L)	5	35
*X*_2_	Glucose (g/L)	5	15
*X*_3_	Tryptone (g/L)	0.5	1.5
*X*_4_	Yeast extract (g/L)	0.5	1.5
*X*_5_	Urea (g/L)	0.5	1.5
*X*_6_	NH_4_Cl (g/L)	5	15
*X*_7_	(NH_4_)_2_SO_4_ (g/L)	5	15
*X*_8_	NaNO_3_ (g/L)	5	15
*X*_9_	K_2_HPO_4_ (g/L)	5	15
*X*_10_	KH_2_PO_4_ (g/L)	5	15
*X*_11_	Tween (g/L)	0.5	1.5
*X*_12_	EDTA (g/L)	0.1	1
*X*_13_	MgSO_4_ (g/L)	0.2	2
*X*_14_	CaCl_2_ (g/L)	0.2	2
*X*_15_	FeSO_4_ (g/L)	0.01	0.05
*X*_16_	MnSO_4_ (g/L)	0.1	0.5
*X*_17_	ZnCl_2_ (g/L)	0.01	0.05
*X*_18_	pH	6	7
*X*_19_	Inoculum size	1 × 10^6^	1 × 10^8^

**Table 2:** Plackett-Burman experimental design matrix for screening of factors affecting xylanase production by *Penicillium* sp.WX-Z1.

No.	*X*_1_	*X*_2_	*X*_3_	*X*_4_	*X*_5_	*X*_6_	*X*_7_	*X*_8_	*X*_9_	*X*_10_	*X*_11_	*X*_12_	*X*_13_	*X*_14_	*X*_15_	*X*_16_	*X*_17_	*X*_18_	*X*_19_	*Y*
1	1	−1	1	1	−1	−1	−1	−1	1	−1	1	−1	1	1	1	1	−1	−1	−1	24.55
2	−1	1	−1	−1	1	1	1	1	−1	1	−1	1	−1	−1	−1	−1	1	1	1	4.30
3	1	1	−1	1	1	−1	−1	−1	−1	1	−1	1	−1	1	1	1	1	−1	−1	20.68
4	−1	−1	1	−1	−1	1	1	1	1	−1	1	−1	1	−1	−1	−1	−1	1	1	8.20
5	−1	1	1	−1	1	1	−1	−1	−1	−1	1	−1	1	−1	1	1	1	1	−1	3.10
6	1	−1	−1	1	−1	−1	1	1	1	1	−1	1	−1	1	−1	−1	−1	−1	1	26.10
7	−1	−1	1	1	−1	1	1	−1	−1	−1	−1	1	−1	1	−1	1	1	1	−1	6.09
8	1	1	−1	−1	1	−1	−1	1	1	1	1	−1	1	−1	1	−1	−1	−1	1	22.81
9	1	−1	−1	1	1	−1	1	1	−1	−1	−1	−1	1	−1	1	−1	1	1	−1	28.50
10	−1	1	1	−1	−1	1	−1	−1	1	1	1	1	−1	1	−1	1	−1	−1	1	1.30
11	1	1	−1	−1	1	1	−1	1	1	−1	−1	−1	−1	1	−1	1	−1	1	−1	22.20
12	−1	−1	1	1	−1	−1	1	−1	−1	1	1	1	1	−1	1	−1	1	−1	1	6.63
13	1	1	1	−1	−1	1	1	−1	1	1	−1	−1	−1	−1	1	−1	1	−1	−1	13.71
14	−1	−1	−1	1	1	−1	−1	1	−1	−1	1	1	1	1	−1	1	−1	1	1	15.11
15	1	1	1	1	−1	−1	1	1	−1	1	1	−1	−1	−1	−1	1	−1	1	−1	25.29
16	−1	−1	−1	−1	1	1	−1	−1	1	−1	−1	1	1	1	1	−1	1	−1	1	3.62
17	−1	1	1	1	1	−1	−1	1	1	−1	1	1	−1	−1	−1	−1	1	−1	−1	6.78
18	1	−1	−1	−1	−1	1	1	−1	−1	1	−1	−1	1	1	1	1	−1	1	1	22.13
19	1	−1	1	1	1	1	−1	−1	1	1	−1	1	1	−1	−1	−1	−1	1	−1	22.71
20	−1	1	−1	−1	−1	−1	1	1	−1	−1	1	−1	−1	1	1	1	1	−1	1	6.18
21	−1	1	−1	1	1	1	1	−1	−1	1	1	−1	1	1	−1	−1	−1	−1	−1	5.98
22	1	−1	1	−1	−1	−1	−1	1	1	−1	−1	1	−1	−1	1	1	1	1	1	22.91
23	1	−1	1	−1	1	1	1	1	−1	−1	1	1	−1	1	1	−1	−1	−1	−1	23.68
24	−1	1	−1	1	−1	−1	−1	−1	1	1	−1	−1	1	−1	−1	1	1	1	1	5.21
25	−1	1	−1	1	−1	1	1	1	1	−1	−1	1	1	−1	1	1	−1	−1	−1	9.18
26	1	−1	1	−1	1	−1	−1	−1	−1	1	1	−1	−1	1	−1	−1	1	1	1	19.71
27	−1	−1	1	−1	1	−1	1	1	1	1	−1	−1	1	1	−1	1	1	−1	−1	8.33
28	1	1	−1	1	−1	1	−1	−1	−1	−1	1	1	−1	−1	1	−1	−1	1	1	20.55
29	−1	−1	−1	1	−1	1	−1	1	1	1	1	−1	−1	1	1	−1	1	1	−1	12.87
30	1	1	1	−1	1	−1	1	−1	−1	−1	−1	1	1	−1	−1	1	−1	−1	1	16.01
31	−1	−1	−1	−1	1	−1	1	−1	1	1	1	1	−1	−1	1	1	−1	1	−1	2.89
32	1	1	1	1	−1	1	−1	1	−1	−1	−1	−1	1	1	−1	−1	1	−1	1	26.11
33	1	−1	−1	−1	−1	1	−1	1	−1	1	1	1	1	−1	−1	1	1	−1	−1	23.65
34	−1	1	1	1	1	−1	1	−1	1	−1	−1	−1	−1	1	1	−1	−1	1	1	5.32
35	1	1	−1	−1	−1	−1	1	−1	1	−1	1	1	1	1	−1	−1	1	1	−1	21.09
36	−1	−1	1	1	1	1	−1	1	−1	1	−1	−1	−1	−1	1	1	−1	−1	1	7.84
37	−1	1	1	−1	−1	−1	−1	1	−1	1	−1	1	1	1	1	−1	−1	1	−1	12.23
38	1	−1	−1	1	1	1	1	−1	1	−1	1	−1	−1	−1	−1	1	1	−1	1	16.65
39	−1	−1	−1	−1	−1	−1	−1	−1	−1	−1	−1	−1	−1	−1	−1	−1	−1	−1	−1	2.82
40	1	1	1	1	1	1	1	1	1	1	1	1	1	1	1	1	1	1	1	29.72

**Table 3:** Effect estimates for xylanase production from the results of Placket-Burman design.

Factors	Medium components	Effect	Standard error	*t*-Ratio	*p*-Value	Confidence level (%)
*X*_1_	Wheat bran	15.74	0.165	95.39	0.0067	99.33
*X*_2_	Glucose	−1.36	0.165	−8.25	0.0767	92.33
*X*_3_	Trypton	−0.12	0.165	−0.70	0.6125	38.75
*X*_4_	Yeast extract	3.05	0.165	18.49	0.0344	96.56
*X*_5_	Urea	−0.54	0.165	−3.29	0.1878	81.22
*X*_6_	NH_4_Cl	−0.78	0.165	−4.72	0.1330	86.7
*X*_7_	(NH_4_)_2_SO_4_	−0.54	0.165	−3.27	0.1891	81.09
*X*_8_	NaNO_3_	5.06	0.165	30.68	0.0207	97.93
*X*_9_	K_2_HPO_4_	−0.52	0.165	−3.16	0.1949	80.51
*X*_10_	KH_2_PO_4_	0.27	0.165	1.65	0.3471	65.29
*X*_11_	Tween	0.54	0.165	3.25	0.1898	81.02
*X*_12_	EDTA	0.39	0.165	2.34	0.2572	74.28
*X*_13_	MgSO_4_	2.35	0.165	14.24	0.0446	95.54
*X*_14_	CaCl_2_	2.16	0.165	13.11	0.0485	95.15
*X*_15_	FeSO_4_	0.77	0.165	4.68	0.1339	86.61
*X*_16_	MnSO_4_	−0.24	0.165	−1.42	0.3897	61.03
*X*_17_	ZnCl_2_	−0.55	0.165	−3.35	0.1846	81.54
*X*_18_	pH	1.88	0.165	11.37	0.0558	94.42
*X*_19_	Inoculum size	0.50	0.165	−3.00	0.2044	79.56

**Table 4:** Box-Behnken experimental design matrix with experimental and predicted values of xylanase production by *Penicillium* sp.WX-Z1.

Runs	Wheat bran (*X*_1_) (g/L)	Yeast extract (*X*_2_) (g/L)	NaNO_3_ (*X*_3_) (g/L)	MgSO_4_ (*X*_4_) (g/L)_4_	CaCl_2_ (*X*_5_) (g/L)	Xylanase (U/mL)
1	5	0.5	10	1.1	1.1	8.52
2	5	1.5	10	1.1	1.1	20.42
3	35	0.5	10	1.1	1.1	33.60
4	35	1.5	10	1.1	1.1	34.76
5	20	1	5	0.2	1.1	21.72
6	20	1	5	2	1.1	24.52
7	20	1	15	0.2	1.1	33.72
8	20	1	15	2	1.1	36.52
9	20	0.5	10	1.1	0.2	26.02
10	20	0.5	10	1.1	2	28.12
11	20	1.5	10	1.1	0.2	30.12
12	20	1.5	10	1.1	2	34.42
13	5	1	5	1.1	1.1	10.72
14	5	1	15	1.1	1.1	18.52
15	35	1	5	1.1	1.1	38.67
16	35	1	15	1.1	1.1	44.72
17	20	1	10	0.2	0.2	23.33
18	20	1	10	0.2	2	28.72
19	20	1	10	2	0.2	28.52
20	20	1	10	2	2	33.52
21	5	1	10	0.2	1.1	10.12
22	5	1	10	2	1.1	15.92
23	35	1	10	0.2	1.1	44.32
24	35	1	10	2	1.1	37.12
25	20	0.5	5	1.1	1.1	21.89
26	20	0.5	15	1.1	1.1	31.99
27	20	1.5	5	1.1	1.1	27.12
28	20	1.5	15	1.1	1.1	37.12
29	20	1	5	1.1	0.2	22.87
30	20	1	5	1.1	2	27.11
31	20	1	15	1.1	0.2	32.12
32	20	1	15	1.1	2	36.34
33	5	1	10	1.1	0.2	11.52
34	5	1	10	1.1	2	15.52
35	35	1	10	1.1	0.2	42.72
36	35	1	10	1.1	2	36.72
37	20	0.5	10	0.2	1.1	25.72
38	20	0.5	10	2	1.1	27.52
39	20	1.5	10	0.2	1.1	29.72
40	20	1.5	10	2	1.1	33.92
41	20	1	10	1.1	1.1	36.12
42	20	1	10	1.1	1.1	38.12
43	20	1	10	1.1	1.1	39.12
44	20	1	10	1.1	1.1	39.67
45	20	1	10	1.1	1.1	40.55
46	20	1	10	1.1	1.1	39.23

**Table 5:** The least-square fit and parameters (significance of regression coefficient).

Model term	Degree of freedom	Estimate	Standard error	*t* Value	*p* > |*t*|
Intercept	1	38.80	0.93	41.53	<0.0001 *
*X*_1_	1	12.59	0.57	22.00	<0.0001 *
*X*_2_	1	2.76	0.57	4.83	<0.0001 *
*X*_3_	1	4.78	0.57	8.35	<0.0001 *
*X*_4_	1	1.26	0.57	2.21	0.0368 *
*X*_5_	1	1.45	0.57	2.54	0.0177 *
*X*_1_*X*_1_	1	−7.55	0.77	−9.74	<0.0001 *
*X*_2_*X*_1_	1	−2.69	1.14	−2.35	0.0272 *
*X*_2_*X*_2_	1	−5.29	0.77	−6.83	<0.0001 *
*X*_3_*X*_1_	1	−0.44	1.14	−0.38	0.7055
*X*_3_*X*_2_	1	−0.03	1.14	−0.02	0.9827
*X*_3_*X*_3_	1	−4.06	0.77	−5.25	<0.0001 *
*X*_4_*X*_1_	1	−3.25	1.14	−2.84	0.0088*
*X*_4_*X*_2_	1	0.60	1.14	0.52	0.6047
*X*_4_*X*_3_	1	0	1.14	0.00	1.0000
*X*_4_*X*_4_	1	−4.96	0.77	−6.40	<0.0001 *
*X*_5_*X*_1_	1	−2.50	1.14	−2.18	0.0385 *
*X*_5_*X*_2_	1	0.55	1.14	0.48	0.6350
*X*_5_*X*_3_	1	−0.005	1.14	−0.00	0.9965
*X*_5_*X*_4_	1	−0.09	1.14	−0.09	0.9328
*X*_5_*X*_5_	1	−4.73	0.77	−6.11	<0.0001 *

* Significant at 5% level (*p* < 0.05).
